# The effect of heart rate variability biofeedback in patients with acute coronary syndrome: A protocol for systematic review and meta-analysis

**DOI:** 10.1097/MD.0000000000032534

**Published:** 2023-01-06

**Authors:** Yuxia Wang, Yinghua Liu, Juan Zeng, Haiying Lu

**Affiliations:** a Department of Cardiology, Huabei Petroleum General Hospital, Hebei, China; b Department of Function, Huabei Petroleum General Hospital, Hebei, China.

**Keywords:** acute coronary syndrome (ACS), autonomic nerve, heart rate variability (HRV) biofeedback, meta-analysis

## Abstract

Acute coronary syndrome (ACS), the acute manifestation of ischemic heart disease, remains a major cause of morbidity and mortality worldwide. Soon after ACS, autonomic imbalance acts to preserve the proper functioning of the cardiovascular system and consequently of the whole body. In this study, we perform a protocol for systematic review and meta-analysis to evaluate the efficacy of heart rate variability biofeedback in improving the prognosis in patients with ACS.

**Methods:**

The protocol of this review was registered in PROSPERO (CRD42022379184). Meanwhile, it will be reported follow the guidelines of the preferred reporting items for systematic reviews and meta-analyses protocol. We will search 3 foreign electronic databases (Cochrane Library, Embase, Pubmed) and 4 Chinese electronic databases (China National Knowledge Infrastructure, WangFang Database, Chinese Biomedical Literature Database and Chinese Scientific Journal Database) to collect potential studies from their inceptions to December 2022. Risk of bias will be assessed according to the Cochrane Risk of Bias Tool. Data synthesis and statistical analysis will be performed using the RevMan 5.3 (The Nordic Cochrane Centre, The Cochrane Collaboration, Denmark) software.

**Results:**

The results of this systematic review will be published in a peer-reviewed journal.

**Conclusion:**

This systematic review will provide high quality evidence to assess the efficacy of heart rate variability biofeedback in patients with ACS.

## 1. Introduction

Acute coronary syndrome (ACS) is a medical emergency. Due to advances in medicine in the past few decades, life expectancy has increased resulting in an aging population in developed and developing countries.^[1]^ ACS causes greater morbidity and mortality in this group of older patients, which appears to be due to age-related comorbidities.

The underlying mechanism of ACS is triggered by atheromatous plaque enlargement, instability, and rupture or erosion.^[2]^ ACS includes ST elevation myocardial infarction, unstable angina and non-ST elevation myocardial infarction.^[3,4]^ Soon after ACS, autonomic imbalance acts to preserve the proper functioning of the cardiovascular system and consequently of the whole body.^[5]^ Nevertheless, the maintenance of this imbalance can lead to harmful consequences in ACS patients.

A relatively easy noninvasive method that is reproducible and cost-effective for estimating cardiac autonomic modulation is evaluation of heart rate variability (HRV), which analyzes variations in the intervals between consecutive normal heart beats or normal R wave peaks, measured in ms.^[6,7]^ Depressed HRV suggests poor functioning of cardiac autonomic modulation and therefore impaired ability of the heart to adapt to a range of physiological and environmental stimuli.^[8]^

The biofeedback of HRV is a technique developed in the late 1980s and early 1990s, systematized and standardized by Lehrer et al,^[9]^ and consists of a psychophysiological training technique in which the subject observes both his respiratory and heart rates on a monitor, in order to try to synchronize the 2 curves until a sinusoidal pattern is obtained, in such a way that a maximum coincidence can be found between the inspiration and the increase of the heart rate, and between expiration and HR decline. Currently, HRV biofeedback is not widely used in ACS patients. In this study, we perform a protocol for systematic review and meta-analysis to evaluate the efficacy of HRV biofeedback in improving the prognosis in patients with ACS.

## 2. Methods

The protocol of this review was registered in PROSPERO (CRD42022379184). Meanwhile, it will be reported follow the guidelines of the preferred reporting items for systematic reviews and meta-analyses protocol.^[10]^ Ethical approval is not required because this review will retrieve publicly available scientific literature.

### 2.1. Inclusion criteria

#### 2.1..1. Type of study.

All randomized controlled trials (RCTs) on the application of HRV biofeedback in patients with ACS will be included with no language limitation. However, animal studies, case reports, case series, commentaries, reviews, non-controlled trials, and other studies that are repeatedly published will be excluded.

#### 2.1..2. Types of participants.

Patients who are diagnosed with ACS will be included, without limits on gender, race, nationality, and medical units.

#### 2.1..3. Types of interventions and comparisons.

Intervention in the experimental group is HRV biofeedback and control group receives usual medical care.

#### 2.1..4. Types of outcome measures.

The primary outcomes are the effective rate, heart rate and blood pressure assessed pre-intervention, post-intervention, and at 1-month follow-up; the secondary outcomes include quality of life and adverse effects.

### 2.2. Search methods

We will search 3 foreign electronic databases (Cochrane Library, Embase, Pubmed) and 4 Chinese electronic databases (China National Knowledge Infrastructure, WangFang Database, Chinese Biomedical Literature Database and Chinese Scientific Journal Database) to collect potential studies from their inceptions to December 2022. The following search terms will be used: HRV biofeedback, ACS and randomized. Search strategy for PubMed is shown in Table 1.

**Table 1:** Search strategy for PubMed.

#1 acute coronary syndrome [Title/Abstract]
#2 myocardial infarction [Title/Abstract]
#3 unstable angina [Title/Abstract]
#4 coronary heart disease [Title/Abstract]
#5 ACS [Title/Abstract]
#6 MI [Title/Abstract]
#7 STEMI [Title/Abstract]
#8 NSTEMI [Title/Abstract]
#9 #1 OR # 2 OR # 3 OR #4 OR #5 OR #6 OR #7 OR #8
#10 heart rate variability biofeedback [Title/Abstract]
#11 HRV biofeedback [Title/Abstract]
#12 biofeedback device [Title/Abstract]
#13 biofeedback system [Title/Abstract]
#14 #10 OR #11 OR #12 OR #13
#15 randomized [Title/Abstract]
#16 randomization [Title/Abstract]
#17 randomized controlled trial [Publication type]
#18 #15 OR #16 OR #17
#19 #9 AND #14 AND #18

ACS = acute coronary syndrome, HRV = heart rate variability, NSTEMI = non-ST elevation myocardial infarction, STEMI = elevation ST myocardial infarction.

### 2.3. Study selection

**Figure 1.**
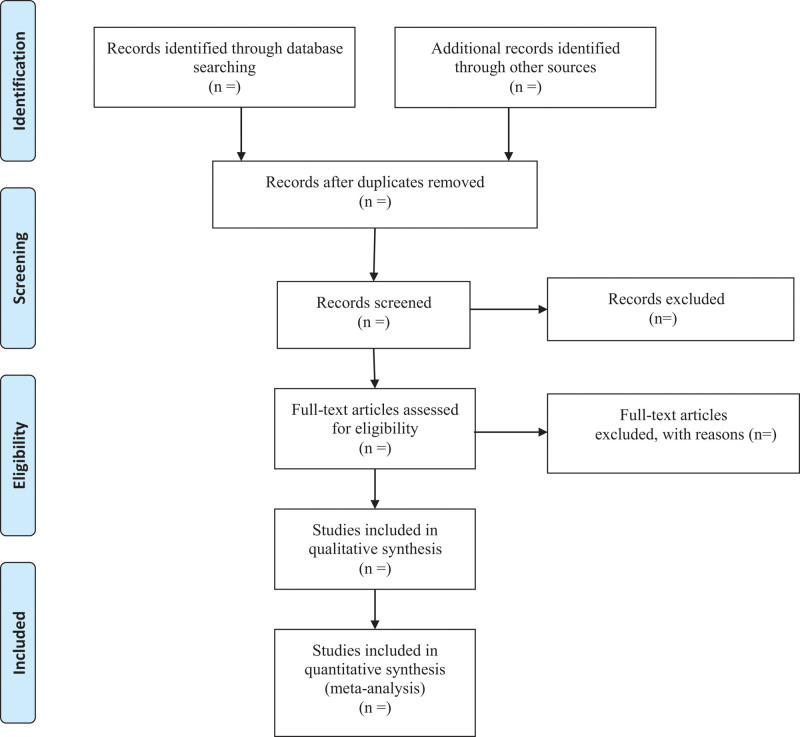
Flow diagram.

Researchers will discuss and determine the screening criteria within the group before searching the studies. The corresponding research members will import the retrieved studies into the document management system of EndnoteX7 for repetition removal. We will then exclude the apparently unqualified literature by reading the headings and abstracts, and determine the final included literature by reading the full text, discussing within the group and contacting the author to know more about the research details. The final list of included studies will be converted to the format of Microsoft Excel. Both the information retrieval and the literature screening will be independently operated by 2 research members. Finally, another research member will resolve the inconsistency and check the final included studies. Study selection is summarized in a PRISMA flow diagram (Fig. 1).

### 2.4. Data extraction

Data extraction will be conducted by 2 researchers using EpiData 3.1 software for double entry. Data for collection include age, sample size, disease diagnosis, combined disease, interventions and details about the control group, follow-up, outcomes, and adverse event. Any disagreement on data collection will be resolved through discussions or negotiations with the third arbitrator. If the data provided in the study are unclear, missing, or presented in a form that is not extractable or difficult to extract reliably, we will contact the author of the study for clarification.

### 2.5. Risk of bias

Risk of bias will be assessed according to the Cochrane Risk of Bias Tool^[11]^ which bases on the following domains: random sequence generation, allocation concealment, blinding of participants and personnel, blinding of outcome assessment, incomplete outcome data, selective outcome reporting, and other sources of bias. Items are scored as low, high, or unclear risk of bias. Two independent researchers will attend the evaluation which will be cross-checked by a third senior 1.

### 2.6. Statistical analysis

Data synthesis and statistical analysis will be performed using the RevMan 5.3 (The Nordic Cochrane Centre, The Cochrane Collaboration, Denmark) software. The standard mean difference with 95% confidence interval will be used to calculate the continuous data, while the dichotomous data will be measured by the rate ratio or odds ratio with 95% confidence interval. For the assessment of heterogeneity, the Chi-squared and *I*^2^ test will be carried out. If there is no significant heterogeneity among studies (*I*^2^ < 50%, *P* > .1), we will use a fixed-effect model, but a random-effects model will be employed if there exists heterogeneity (*I*^2^ ≥ 50%, *P* < .1).

### 2.7. Assessment of reporting biases

We will conduct analysis of Egger publication bias plot and Begg funnel plot with pseudo 95% confidence limits to determine the publication bias in all the literature with sufficient studies (more than 10 trials).^[12]^

### 2.8. Quality of evidence

Grading of Recommendations Assessment, Development, and Evaluation (GRADE)^[13]^ will be used to assess the results. In the GRADE system, the quality of evidence will be categorized into 4 levels: high, moderate, low, and very low quality.

## 3. Discussion

Multiple clinical studies have shown that HRV biofeedback can alleviate neurocardiac dysfunction and improve clinical outcomes in neuropsychiatric and cardiovascular disorders,^[14–16]^ possibly mediated by augmented respiratory sinus arrhythmia triggering increased baroreflex gain and parasympathetic outflow.^[17,18]^ This is the first meta-analysis to evaluate the efficacy of HRV biofeedback in patients with ACS. Some limitations of this study should be noted: Our studies only search database in English and Chinese because of language barriers. So, there may exist a language bias. We will do a full-scale in the future to evaluate it better. The large clinical heterogeneity may exist for different disorder stage, duration of intervention and action consistency, future high quality RCTs are required to confirm the conclusion.

## Author contributions

**Investigation:** Juan Zeng.

**Methodology:** Haiying Lu.

**Writing – original draft:** Yuxia Wang.

**Writing – review & editing:** Yinghua Liu.
